# Size and surface-energy dependence of the adsorption/desorption equilibrium in ethanol electro-oxidation by Pd-nanoparticles. Theory and experiment†

**DOI:** 10.1039/d1ra08742h

**Published:** 2022-01-18

**Authors:** J. Maya-Cornejo, S. I. Hernández, Miriam Estévez, I. Santamaría-Holek

**Affiliations:** Unidad Multidisciplinaria de Docencia e Investigación-Juriquilla, Facultad de Ciencias, Universidad Nacional Autónoma de México Juriquilla Querétaro 76230 Mexico iqm_jamc@yahoo.com.mx isholek@ciencias.unam.mx; Centro de Física Aplicada y Tecnología Avanzada, Universidad Nacional Autónoma de México Boulevard Juriquilla 3001 76230 Santiago de Querétaro Qro Mexico

## Abstract

The relation between current and voltage in the electro-oxidation of ethanol by metal nanoparticles depends on experimental parameters like the applied potential, peak potential, temperature, the electron-transfer coefficient, and the number of molecules adsorbed at active sites on the nanoparticle surface. In this form, the oxidation current depends on the ability of the nanoparticles to adsorb the ethanol molecules. Though the Laviron model well describes this phenomenology, few studies focus on the dependence of the oxidation current on the size and surface properties of the metal nanoparticles. Here, we present an experimental and theoretical study that comprises the synthesis of palladium-based nanoparticles and the generalization of the Laviron model that allows determining the dependence of the oxidation current on the size, surface energy, and adsorption–desorption properties of the nanoparticles for the ethanol oxidation. The determination of the adsorption–desorption equilibrium and the electro-oxidation current dependence with the physicochemical properties of the materials was carried out by electrochemical characterization.

## Abstract 2

The current–voltage relation of ethanol electro-oxidation by Pd nanoparticles depends on their radius, the temperature and electron-transfer coefficient, the adsorption equilibrium constant, the surface energy, and the number of active surface sites.

## Graphical abstract

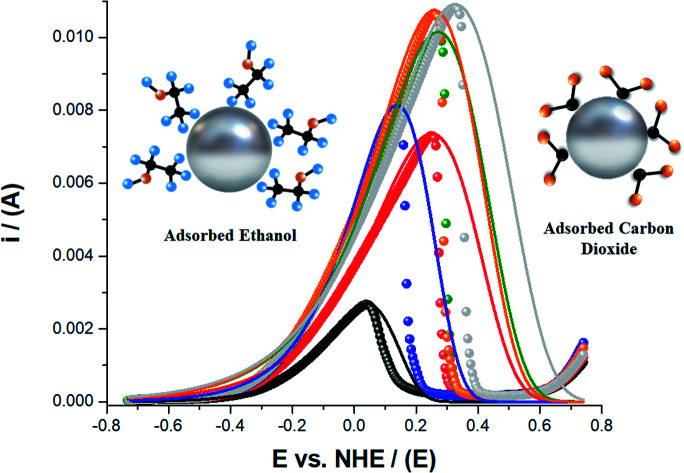

## Introduction

In order to decrease the dependence on the use of Pt-based materials on electrochemical processes, an alternative and viable option is its replacement by Pd-based materials, because of their unique electrocatalytic properties in alkaline media for the electro-oxidation of organic molecules, such as ethanol.^1–3^ Furthermore, the development of Pd-based electrocatalysts has been focused on their high electrocatalytic activity, the synthesis of stable nanoparticles,^4^ and generation of specific morphologies.^4–8^ However, for electrochemical reactions, one of the most critical steps is the adsorption of the species on the electrocatalytic surface. This adsorption depends directly on the intrinsic characteristics of the materials, their chemical compositions, their crystallographic arrays, their size, the concentrations of the species in the bulk dissolution, and on the surface–electrolyte interface.^9–13^

The oxidation current depends on the number of adsorbed fuel molecules, and the adsorption of molecules on a surface is related to the energy associated with the electrochemical process. A useful theoretical model that takes into account such characteristics and describes the production of the oxidation current is the Laviron equation^14^1
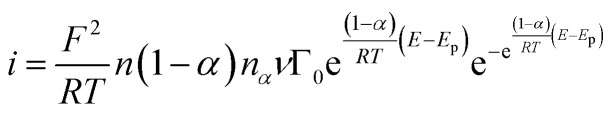


This equation describes the dependence of the oxidation current (*i*) on the number of adsorbed molecules (*Γ*_0_), the applied potential (*E*) and the peak potential (*E*_P_). Also, in the Laviron equation appear the Faraday's constant (*F*), ideal gas constant (*R*), temperature (*T*), number of electrons involve in the reaction (*n*), transfer coefficient array ((1 − *α*)*n*_*α*_) and sweep velocity (*v*). Underlying the deduction of the Laviron equation is the assumption that the adsorption process is not the control step.^14^

In the case of electrocatalytic materials, the mathematical description of the adsorption process must include the geometry of the electrocatalytic nanoparticles since such geometry may influence the catalytic efficiency. In this work, we reconsider the Langmuir–Hinshelwood adsorption–desorption reaction in the presence of a curved surface. The dependence of the chemical affinity of adsorption on the particle geometry enters through an activity coefficient that depends on the particle radius, according to the classical Gibbs–Thompson approach. The corrections to the Laviron equation incorporate the explicit dependence of the current on the particle size, the surface energy, and the equilibrium constant for the ethanol adsorption–desorption process.^15,16^

## Experimental

### Chemicals

Polyvinylpyrrolidone (PVP, mol wt 40 000, Sigma-Aldrich), ethylene glycol (EG, 99.8%, Sigma), ascorbic acid (AA, 99.0%, Sigma-Aldrich), potassium tetrachloropalladate(ii) (K_2_PdCl_4_, 99%, Sigma-Aldrich), Vulcan carbon XC-72R (Cabot), ethanol (96%, industrial grade) for the dispersion of the carbon support, ethanol (99.7%, Sigma-Aldrich) as fuel, Nafion® (5 wt% in isopropanol, Sigma-Aldrich), potassium chloride (99.7%, Sigma-Aldrich) to saturated the reference electrode dissolution and potassium hydroxide (KOH 85%, Sigma-Aldrich) were used as received.

### Synthesis of Pd/C electrocatalysts

The Pd/C electrocatalysts were synthesized employing a chemical reduction method. The PVP, as a surfactant and 10 mL EG as reaction media, were placed in a round-bottom flask connected to a condenser at a stirring constant. The temperature raised around 80 °C. After the PVP became dissolved in the EG, 0.06 g of K_2_PdCl_4_ and 0.127 g of ascorbic acid were added. The palladium reduction reaction was maintained under constant stirring at 80 °C for 90 minutes. Simultaneously, 60 mg of Vulcan carbon were dissolved in 50 mL ethanol and stirred for 30 minutes to disperse the Vulcan carbon. After this step and when the reduction of the palladium was carried out, the synthesis dissolution was added drop by drop into the dispersed Vulcan carbon. The resulting powder was removed several times with distilled water and dried at room temperature overnight.

### Synthesis of Cu@Pd/C electrocatalysts

The synthesis of Cu@Pd nanoparticles was carried out using previously reported by our group.^17^ In summary, 0.1 g PVP, used as a surfactant, was added to a round-bottom flask containing 10 mL EG as a reaction medium. Then, the solution was heated to 80 °C with continuous magnetic stirring. At this temperature 0.06 g of CuSO_4_ was added as precursor salt. Later, the Cu^0^ core nanoparticle was formed after the addition of 0.127 g of AA as the reducing agent. The reaction media was maintained under agitation for 30 min. Then, 28 mg K_2_PdCl_4_ and AA dissolved separately in 1 mL of distilled water and placed in the flask. The resulting solution was stirred for 90 min to complete the redox replacement reaction between Cu^0^ and Pd^2+^ atoms. Before the reduction reaction finished, 50 mL ethanol with 56 mg of Vulcan carbon as support was placed in the flask, maintaining the stirring for 30 min, and after this time, the dissolution reduction reaction was dropped in. Then, the electrocatalytic materials were washed several times with distilled water and dried at room temperature overnight.

### Electrochemical characterization

The electrochemical profiles and the electrocatalytic activity for the ethanol oxidation reaction (EOR) in alkaline media were carried out with the linear sweep voltammetry and cyclic voltammetry as electrochemical techniques using a BioLogic VSP Potentiostat/Galvanostat. The electrochemical data were captured using a three-electrode electrochemical cell configuration. A glassy carbon (Basi®, 0.0769 cm^2^) served as a working electrode, a calomel electrode saturated with KCl (SCE) was the reference electrode, and a graphite rod was the counter electrode. The catalytic inks, used to test the materials in alkaline media and ethanol, were prepared mixing 1 mg catalytic powder, 73 μL isopropanol as the dispersant and 7 μL Nafion® as a binder. After the ink was stirring, it was deposited dropwise in the working electrodes using 10 μL to cover the entire glassy carbon electrode zone. The electrochemical profiles were obtained using 0.6 M of KOH dissolution as an electrolyte in the absence of ethanol, while the EOR tests were carried out using 1 M of KOH dissolution and varying the ethanol concentration (from 0.5 to 3 M). Cyclic voltammograms were obtained in a potential range from −0.7 to 0.8 V *vs.* NHE (normal hydrogen electrode = SCE + 0.241 V). The scan rate was 50 mV s^−1^ for the electrochemical profiles and 20 mV s^−1^ for the electrocatalytic evaluation. Before each experiment, the electrolytic solutions were bubbled with nitrogen gas for 10 minutes (Infra, 99.999%).

## Results

### Modified Laviron equation

The modification of the Laviron equation focuses on the number of adsorbed molecules. A Langmuir-like model containing different physicochemical terms helps us incorporate the physicochemical and morphological characteristics of the materials in the adsorption process description.

The first step before the electrochemical oxidation reaction starts is the adsorption reaction between the molecules of alcohol in the solution and the surface of the nanoparticles. At the equilibrium, this adsorption reaction is:2
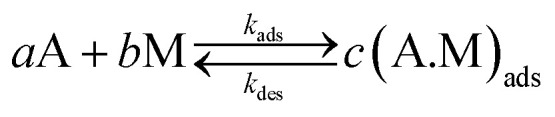
where A represents the alcohol molecules in solution, M the active sites at the surface of the nanoparticles, (A.M)_ads_ the molecules of alcohol adsorbed, and *a*, *b* and *c* are the stoichiometric coefficients. As it is well known, the adsorption reaction can be represented in terms of the chemical potentials for each involved species and the affinity 
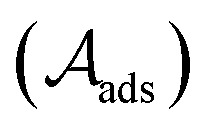
. For the adsorption of ethanol over the metal surface, it can be assumed that each ethanol molecule binds over a single active site and, therefore, the stoichiometric coefficients of reaction (2) can be assumed as equal to the unity. Using this, the chemical affinity of the reaction is given by the expression3
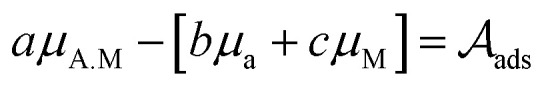


This relation is relevant since the chemical potentials in (3) can be rewritten by their definition in terms of the molar fractions *x*_A.M_ of each species: *μ*_i_ = *μ*^0^_i_ + *RT* ln *x*_i_, where *μ*^0^_i_ is the standard chemical potential. After grouping some terms, we obtain4
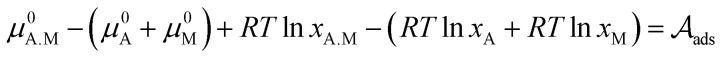
Here, we will use the fact that the difference of standard chemical potentials Δ*μ*_0_ ≡ *μ*^0^_A.M_ − (*μ*^0^_A_ + *μ*^0^_M_) depends on the geometry of the catalyzer, that is, on the radius of the nanoparticles. The explicit expression for Δ*μ*_0_ can be determined by using the Gibbs–Thomson equilibrium relation^18^ between the chemical potential and the surface energy of the particle:5
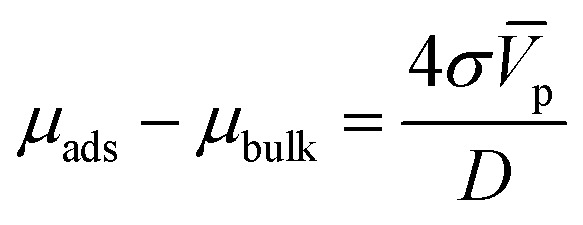
Here *σ* is the surface energy of the nanoparticle and *V̄*_p_ and *D* their molar volume and diameter, respectively. For the adsorption reaction, the chemical potential associated with the adsorbed species on the catalyst surface is defined by *μ*_ads_, while the sum of chemical potentials associated with the alcohol in the bulk of the dissolution and the surface of the catalyst can be described by *μ*_bulk_. Therefore, the Gibbs–Thomson equilibrium can be written in the form:6
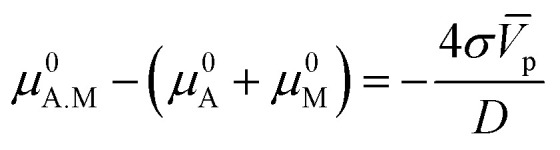


After substitution of eqn (6) into (4) and making some rearrangements we obtain7
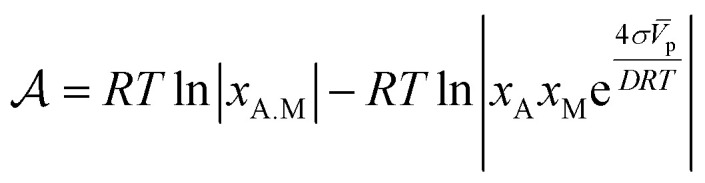


The kinetic law for adsorption can now be obtained by making a series expansion of the natural logarithm: ln|*y*| ≃ *y* − 1 + O(*y*^2^). Then, the affinity reduces to8
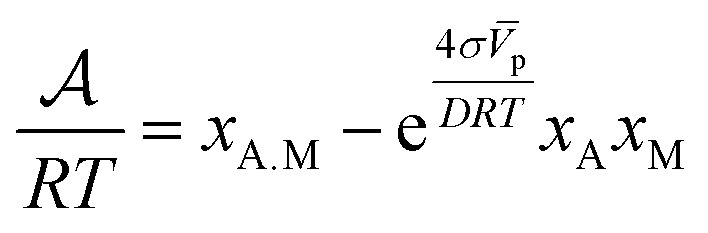


Since the velocity of the reaction is proportional to the negative of the chemical affinity (8),^19^ then it can be shown that the kinetics of the adsorption can be expressed in the form9
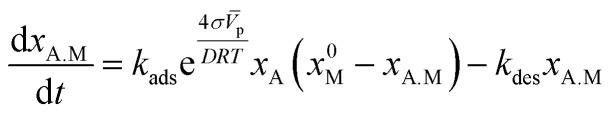
where we have used the fact that *x*_M_ = *x*^0^_M_ − *x*_A.M_ is the effective number of free active sites at time *t* from a total initial number of *x*^0^_M_. At equilibrium, from the previous equation we find the following expression for the molar fraction of the adsorption isotherm of alcohol molecules10
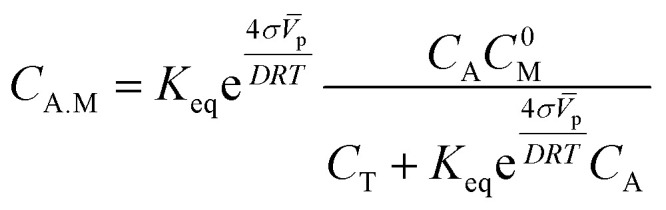
where we have introduced the equilibrium constant for the adsorption–desorption reaction of alcohol, *K*_eq_ = *k*_ads_/*k*_des_, on the surface of the particles. Additionally, we have used the molar fraction definition *x*_i_ =*c*_i_/*c*_T_ to write the isotherm as a function of mole number concentrations. Eqn (10) relates the concentration of adsorbed alcohol molecules (*C*_A.M_) with their concentration in the dissolution (*C*_A_). It also includes the initial concentration of active sites (*C*^0^_M_) and the total concentration (*C*_T_). The *C*_A.M_ depends on the particle size (*D*), the surface energy (*σ*), the molar volume (*V̄*_p_), and the temperature (*T*).

The concentration of the adsorbed molecules (*C*_A.M_) in (10) allows rewriting the number of adsorbed molecules (*Γ*_0_) in order to obtain the modified Laviron equation11
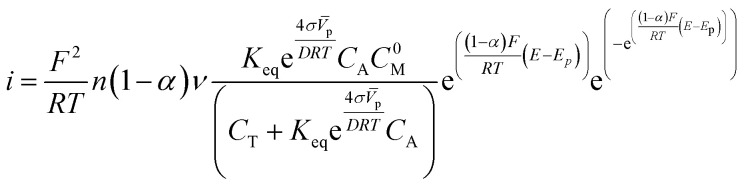


Furthermore, in the previous equation, we have determined that the term (1 − *α*)*n*_*α*_ can be substituted by (1 − *α*), because the factor *n*_*α*_ has directly depended on *α*, thus resulting in a shift of *α* values. This reduces the calculation of additional variables that increase the theoretical error.

We made some considerations to calculate the parameters to use in the modified Laviron equation. The total concentration of our system is the sum of the alcohol concentration and the initial metal concentration, and is given by the following relationship *C*_T_ = *C*_A_ + *C*^0^_M_, obtaining a new arrangement for the modified Laviron equation:12
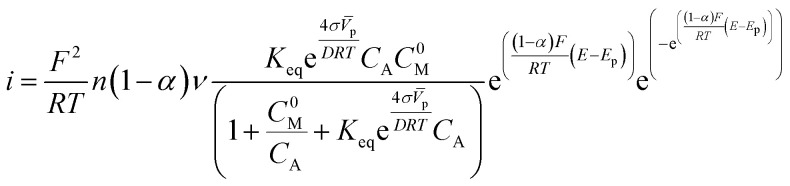


However, the metal concentration must be the initial concentration of atoms or active sites on the metal surface, which is given by13
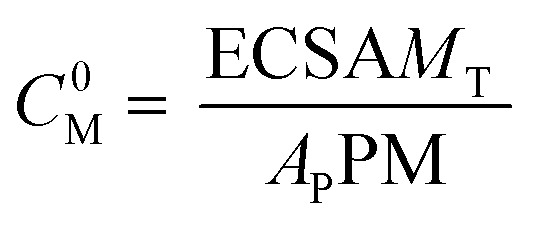
where *M*_T_ is the total weight of the metal in the working electrode, *A*_P_ is the particle area and ECSA is the electrochemical surface area calculated by integrating the charge needed to reduce the Pd oxides formed (*Q* units: μC), assuming that a theoretical charge of 405 μC cm^−2^ is required to reduce a full monolayer of Pd oxides.^20^

### Validation of the modified Laviron equation

To probe the modified Laviron equation, we calculated the amount of adsorbed molecules and compared it with the Laviron equation results. According to the modified Laviron eqn (12), the oxidation current depends on the particle size and the equilibrium constant of the adsorption reaction. It also depends on the surface energy and the concentration of active sites, which depend on the particle size.

We developed a program in a free-language programing (Octave) that runs iteration-sequences to calculate the electron-transfer coefficient (*α*) and the amount of adsorbed molecules (*Γ*_0_) for the Laviron equation. For the modified Laviron equation, the iteration-sequences calculate the adsorption equilibrium constant (*K*_eq_) and the electron-transfer coefficient (*α*). To validate, the modified Laviron equation was tested with two different methods.

The first was an implicit method, in which we have considered an initial calculation of the number of adsorbed molecules and the value of the electron-transfer coefficient for the Laviron equation. Then, with the previous results, we calculated the adsorption equilibrium constant (*K*_eq_) and the number of adsorbed molecules (*Γ*_0_) for the modified Laviron equation.

The second was an explicit method, in which we used eqn (12) to calculate the value of the electron-transfer coefficient (*α*) directly in the modified Laviron equation to obtain the adsorption equilibrium constant (*K*_eq_) and, finally, the calculation of the number of adsorbed molecules (*Γ*_0_).

The results of the two methods were compared between them to determine if our modification of the Laviron equation is consistent with the original Laviron equation.

The results for the electron-transfer coefficient, the amount of adsorbed molecules and the constant equilibrium of adsorption will be presented as Table S1 in the ESI.†

The program evaluation used reported experimental parameters of the catalysts used to obtain the unknown variables. In Table S2† we report the values of the parameters used for fitting the electrochemical response of the Pd/C and Cu@Pd/C nanoparticles during the electro-oxidation of ethanol at different concentrations. Furthermore, we considered the surface energy of the palladium of 0.684 J m^−2^ for the crystallographic plane (111)^21^ because, in previous works, we determined that was the predominant plane even though the composition of materials was Pd/C and Cu@Pd/C.^17,21–23^

**Fig. 1:**
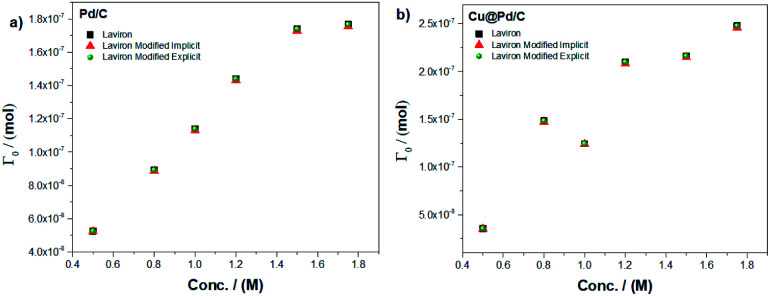
Amount of the adsorbed molecule as a function of the ethanol concentration for (a) Pd/C and (b) Cu@Pd/C catalysts.

We used the Laviron eqn (1) and the modified Laviron eqn (12) to calculate the amount of adsorbed molecules for each catalyst. For the Pd/C catalyst, the results of the amount of molecules adsorbed using the implicit method between Laviron and modified Laviron equations show an increase when the concentration of ethanol increases (Fig. 1a). Furthermore, using the explicit method, the number of molecules adsorbed for the modified Laviron equation was similar compared to the Laviron equation. The results of the amount of molecules adsorbed for the Cu@Pd/C catalyst show similar values using both the explicit and implicit methods, and a consistent increasing behaviour when the concentration of ethanol increases (Fig. 1b).

Fig. 1 shows that the modified Laviron eqn (12), that includes the particle diameter, the adsorption reaction constant, the surface energy, the concentration of the alcohol and the initial concentration of active sites, allows for fittings of the data as accurate as the original Laviron eqn (1). The advantage is that the new formula establishes the correlations between the current values with the above-mentioned physiochemical properties of the particles. The increase in the number of molecules adsorbed with the concentration of ethanol for both catalysts was expected due to the molecules of ethanol increase near to the active sites over the nanoparticles to promote their adsorption.

**Fig. 2:**
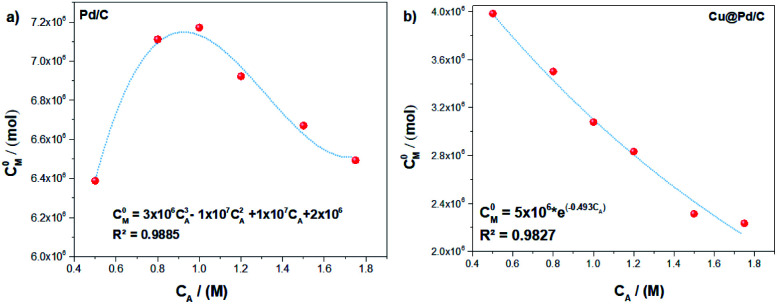
Tendency adjustment of the initial active sites concentration as a function of the concentration for the (a) Pd/C and (b) Cu@Pd/C catalysts.

However, as we have mentioned in the eqn (13), the initial concentration of active sites (*C*^0^_M_) depends on the electrochemical surface area (ECSA). It exhibits a direct relation with the ethanol concentration, showing a nonlinear behaviour as a function of the ethanol concentration (Fig. 2). The tendency of the (*C*^0^_M_) values for the Pd/C catalyst shows a non-monotonic behaviour while the ethanol concentration increases, and the mathematical function that describe it is a cubic polynomial equation (Fig. 2a). Furthermore, the Cu@Pd/C catalyst values of (*C*^0^_M_) exhibit a weak exponential decrease tendency, while the ethanol concentration increases, see (Fig. 2b). These results were expected because the concentration of active sites decreases when the amount of molecules in solution increases, promoting the adsorption of alcohol on the nanoparticles. This analysis revels that the adsorption process is different for each catalyst despite the fact that both of it have Pd on the surface, suggesting that the surface energy changes due to the presence of Cu.

**Fig. 3:**
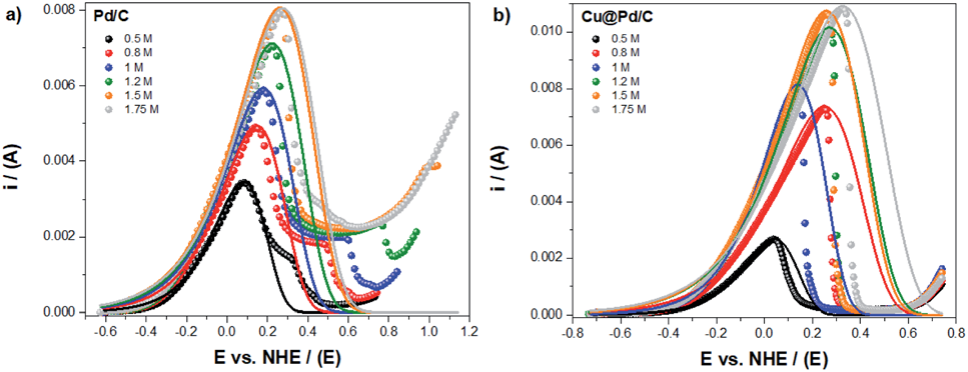
Experimental (dots) and theoretical (line) electro-oxidation of ethanol for the (a) Pd/C and (b) Cu@Pd/C catalysts.

The modified Laviron equation with the correction of the initial active sites concentration was used to obtain the theoretical fitting for the electro-oxidation of ethanol and compared with the experimental results (Fig. 3). The fitting of the electrochemical oxidation for the Pd/C catalyst shows that the experimental and theoretical results have an excellent correlation with the equilibrium potential until the peak potential (Fig. 3a). After that, if the shape of the peak is symmetric (taking as reference the value of peak potential), the fitting continues with a good correlation. However, a deviation from experiments and theoretical results is appreciable when the potential shifts to higher values (in comparison with the peak potential) by the formation of a shoulder after which a sudden decrease of the experimental currents is observed. This fact suggests poisoning effects due to the presence of an excess of adsorbed by-products from the ethanol oxidation reaction.

**Fig. 4:**
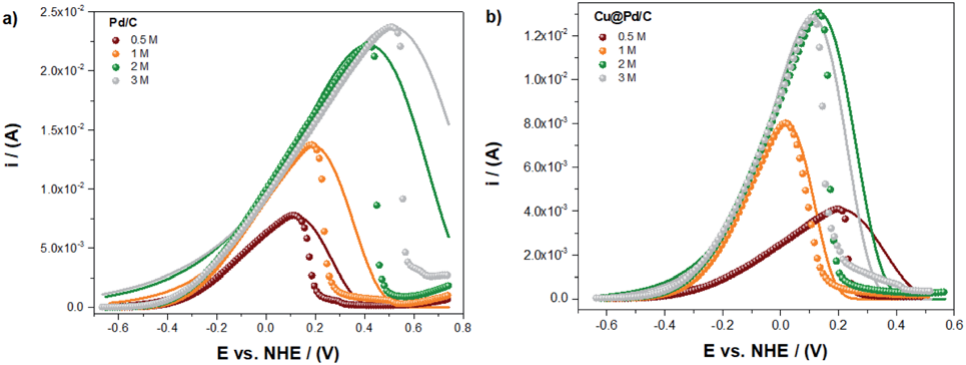
The numerical fitting (lines) obtained with the modified Laviron eqn (12), and the experimental values (dots), for the electro-oxidation of ethanol for the (a) Pd/C and (b) Cu@Pd/C catalysts.

For the Cu@Pd/C catalyst, in Fig. 4b, the experimental and theoretical results present a good correlation between the equilibrium and peak potential. After that, the current shows an abrupt decrease in their values, suggesting that the adsorption process is not the leading contribution (Fig. 3b). This behaviour can be related to the strong adsorption of the by-products from the electro-oxidation of ethanol that covered active sites avoiding the adsorption of alcohol molecules and, therefore, stopping the electro-oxidation of ethanol. Both behaviors are known as the poisoning of the catalysts. It is thus worth mentioning that the Laviron equation does not consider this effect explicitly. The results obtained here show a limitation in the Laviron approach due to the lack of considering the strong adsorption of the by-products of the alcohol electro-oxidation that block the adsorption of new alcohol molecules on the active sites, resulting in surface poisoning.

The limitation of the Laviron approach related to the oxidation peak shape was corroborated at higher concentrations of ethanol (Fig. 4). The numerical fitting of the experimental currents shows that the lower error (*R*^2^ = 0.98174) corresponds to the oxidation of ethanol 1 M using the Cu@Pd/C catalyst, because the peak presents a symmetric shape with respect to the potential peak value (Fig. 4b). For all the other compositions, the peaks exhibit a negative asymmetry, which in some cases are lower and in other cases are higher with respect to the peak potential value (Fig. 4a and b). Therefore, we may conclude that if the catalysts shows a poisoning effect due to the presence of by-products of ethanol electro-oxidation, the Laviron approach is not accurate enough to describe the current–potential curve.

For potentials larger than the peak potential, poisoning processes occur in many Pd-based materials^17,24–29^ and the Laviron model fails to describe the experimental current behaviour. This failure is because the Laviron model does not consider explicitly the multi-step reaction for the ethanol oxidation.

**Fig. 5:**
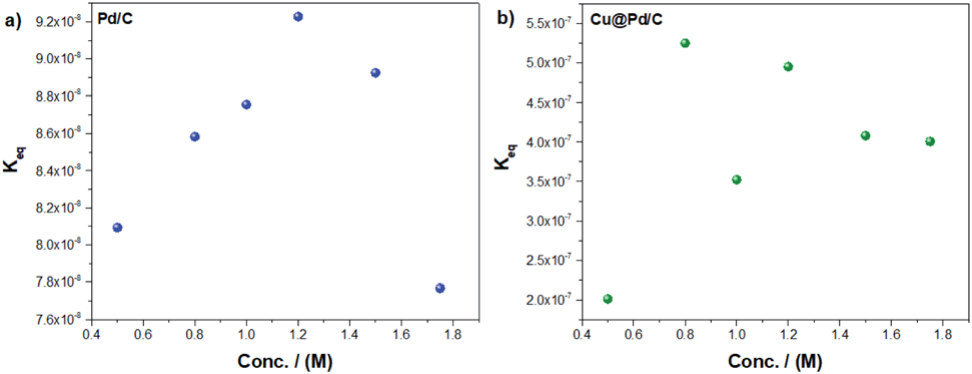
Equilibrium adsorption constant as a function of the ethanol concentration for the (a) Pd/C and (b) Cu@Pd/C catalysts.

The modified Laviron equation includes the equilibrium adsorption constant as a vital parameter to understand the adsorption process of the molecule of alcohols over the active sites (Fig. 5). For the Pd/C catalyst, the equilibrium adsorption constant rises while the ethanol concentration increases until 1.2 M. However, for higher concentrations, their values decrease (Fig. 5a). As previously mentioned, this behaviour seems associated with the blocking of active sites of the nanoparticles with the by-products. Furthermore, the Cu@Pd/C catalyst (Fig. 5b) shows a similar behaviour as the Pd/C catalyst. The values of *K*_eq_ increase until 0.8 M of ethanol but from the concentration of 1 M of ethanol, the results begins to decrease. This behavior also supports the poisoning effect previously discussed (Fig. 3b and 4b).

## Conclusions

In this work, we propose a modified Laviron equation to describe the adsorption-controlled oxidation currents in nanoparticle-based electrodes. The new equation incorporates meaningful geometrical and physicochemical parameters such as the particle diameter, the adsorption reaction constant, the surface energy, and the initial concentration of active sites. With these variables, the equation allows an excellent theoretical approximation of experiments, resulting in a reliable interpretation because of its good performance in the fittings of the data.

However, after the peak potential, the modified Laviron equation shows a limitation regarding with the strong adsorption of the by-products from the ethanol electro-oxidation process that covers the active sites, avoiding the ethanol adsorption and halting their electro-oxidation reaction. The result is the behaviour that we know as catalyst's poisoning.

Furthermore, the amount of the initial active sites as a function of the ethanol concentration reveals that the adsorption process is different for each catalyst despite having Pd on the surface, suggesting that the presence of Cu exhibits a critical effect on the surface. Finally, the adsorption reaction equilibrium constant corroborates the poisoning of the nanoparticles surface due to the strong adsorption of the by-products from the ethanol electro-oxidation.

## Author contributions

JMC: conceptualization, formal analysis, funding acquisition, investigation, methodology, software, visualization, writing – original draft. SIH: writing – review & editing, methodology, supervision. ME: writing – review & editing. ISH: conceptualization, funding acquisition, investigation, methodology, supervision, writing – original draft.

## Conflicts of interest

There are no conflicts to declare.

## Supplementary Material

RA-012-D1RA08742H-s001
