# Supplementary material for: Size and surface-energy dependence of the adsorption/desorption equilibrium in ethanol electro-oxidation by Pd-nanoparticles. Theory and experiment
Source: RSC Adv. 2022 Jan 18;12(4):2525–30. doi: 10.1039/d1ra08742h (PMC8979014; doi:10.1039/d1ra08742h)
Supplement: RA-012-D1RA08742H-s001 [file RA-012-D1RA08742H-s001.pdf]

Electronic Supplementary Material (ESI) for RSC Advances.
This journal is © The Royal Society of Chemistry 2022

Supplementary Information

Size and surface-energy dependence of the adsorption/desorption equilibrium in ethanol electro-oxidation by Pd-nanoparticles. Theory and experiment.

J. Maya-Cornejo[a]*, S. I. Hernández[a], Miriam Estévez[b] and I. Santamaría-Holek[a]*

[a] Unidad Multidisciplinaria de Docencia e Investigación-Juriquilla, Facultad de Ciencias, Universidad Nacional Autónoma de México, Juriquilla, Querétaro 76230, Mexico.

[b] Centro de Física Aplicada y Tecnología Avanzada, Universidad Nacional Autónoma de México, Boulevard Juriquilla 3001, 76230 Santiago de Querétaro, Qro, México.

Physiochemical characterization

The morphological and structural characterization of the samples was carried out using scanning electron microscopy (SEM) and scanning/transmission electron microscopy (STEM). SEM images were obtained using a Hitachi SU8230 cold field emission (CFE) SEM/STEM microscope at 30 keV accelerating voltage at a distance of 8 mm with the Z contrast STEM.

Scanning transmission electron microscopy (STEM)

The particle size of the materials is an essential parameter for the modified Laviron equation to determine the oxidation current. For the Pd/C catalyst, the micrographs show nanoparticles with a quasi-spherical morphology (Fig. 1a). Furthermore, the particle size presents values around 3 nm according to the histogram obtained (Fig. 1c). The Cu@Pd/C catalyst also shows semi-spherical morphology (Fig. 1b). The particle size is around 5 nm (Fig. 1d). The modified Laviron equation considers a spherical shape for the particles, and those results are according to our suggestion.

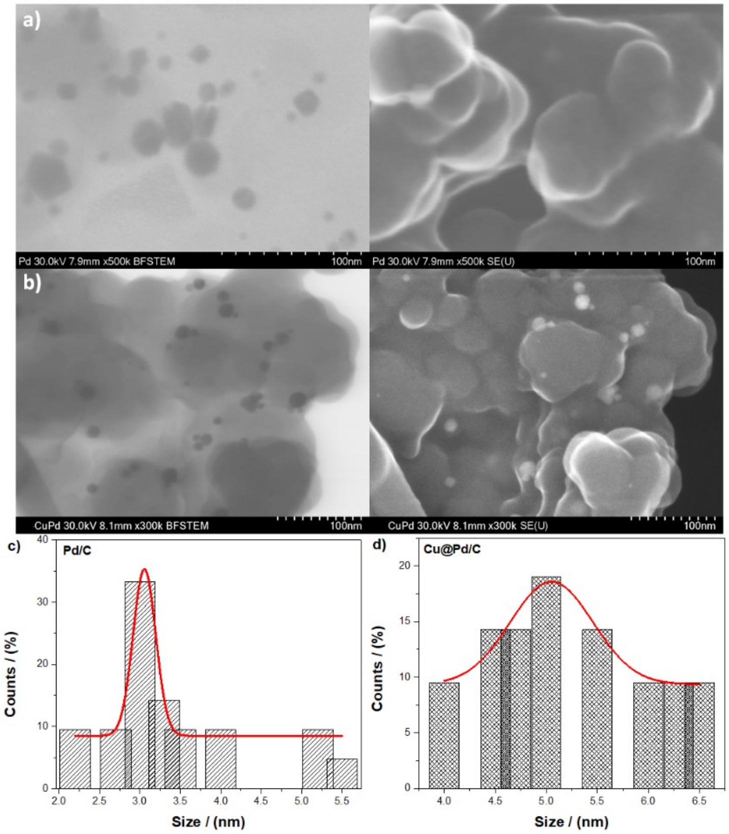

Figure S1.- Micrographs for the a) Pd/C and b) Cu@Pd/C catalysts and their histogram of particle size for the c) Pd/C and d) Cu@Pd/C, respectively.

Table S1.- Numerical results for the parameters of Laviron and modified Laviron equation.

**Sample**	**Conc. (M)**	**Laviron equation**		**Modified Laviron equation**		
		Γ_0	α	Γ_0	K_{eq}	α
**Pd/C**	0.5	5.26x10^{-8}	0.8024	5.234x10^{-8}	8.056x10^{-8}	0.8024
	0.8	8.92x10^{-8}	0.8335	8.873x10^{-8}	8.537x10^{-8}	0.8335
	1	1.138x10^{-7}	0.8430	1.131x10^{-7}	8.706x10^{-8}	0.8430
	1.2	1.439x10^{-7}	0.8512	1.43x10^{-7}	9.173x10^{-8}	0.8512
	1.5	1.739x10^{-7}	0.8606	1.728x10^{-7}	8.867x10^{-8}	0.8606
	1.75	1.766x10^{-7}	0.8629	1.755x10^{-7}	7.717x10^{-8}	0.8629
**Cu@Pd/C**	0.5	3.551x10^{-8}	0.7724	3.55x10^{-8}	2.012x10^{-7}	0.7724
	0.8	1.483x10^{-7}	0.8507	1.483x10^{-7}	5.252x10^{-7}	0.8507
	1	1.244x10^{-7}	0.8030	1.233x10^{-7}	3.5x10^{-7}	0.8030
	1.2	2.098x10^{-7}	0.8544	2.098z10^{-7}	4.953x10^{-7}	0.8544
	1.5	2.162x10^{-7}	0.8504	2.162x10^{-7}	4.082x10^{-7}	0.8504
	1.75	2.477x10^{-7}	0.8674	2.477x10^{-7}	4.0095x10^{-7}	0.8674

Table S2.- Experimental parameters for feeding the iteration program for the electro-oxidation of ethanol.

**Sample**	**Conc. (M)**	**Electron number**	**Sweep velocity (mV s^{-1})**	**Half-peak potential (V)**	**Half-peak current (A)**	**Peak potential (V)**	**Peak current (A)**
**Pd/C**	0.5	12	20	-0.1288	0.001535	0.0815	0.00343
	0.8	12	20	-0.1016	0.0022	0.1472	0.0049
	1	12	20	-0.0749	0.00277	0.1792	0.00589
	1.2	12	20	-0.0676	0.00299	0.2232	0.007058
	1.5	12	20	-0.05794	0.00326	0.2611	0.00799
	1.75	12	20	-0.0493	0.003256	0.2751	0.007979
**Cu@Pd/C**	0.5	12	20	-0.1334	0.00128	0.0394	0.00267
	0.8	12	20	-0.0205	0.00338	0.250	0.00730
	1	12	20	-0.0718	0.00363	0.1381	0.00809
	1.2	12	20	-0.0178	0.00444	0.2704	0.01007
	1.5	12	20	-0.0166	0.004806	0.259	0.01066
	1.75	12	20	0.0198	0.00498	0.326	0.01082
